# Debunking (the) Retribution (Gap)

**DOI:** 10.1007/s11948-019-00148-6

**Published:** 2019-10-16

**Authors:** Steven R. Kraaijeveld

**Affiliations:** Wageningen University & Research, Wageningen, The Netherlands

**Keywords:** Retribution, Retribution gaps, Human–robot interactions, Debunking arguments, Moral intuitions, Moral responsibility

## Abstract

Robotization is an increasingly pervasive feature of our lives. Robots with high degrees of autonomy may cause harm, yet in sufficiently complex systems neither the robots nor the human developers may be candidates for moral blame. John Danaher has recently argued that this may lead to a retribution gap, where the human desire for retribution faces a lack of appropriate subjects for retributive blame. The potential social and moral implications of a retribution gap are considerable. I argue that the retributive intuitions that feed into retribution gaps are best understood as deontological intuitions. I apply a debunking argument for deontological intuitions in order to show that retributive intuitions cannot be used to justify retributive punishment in cases of robot harm without clear candidates for blame. The fundamental moral question thus becomes what we ought to do with these retributive intuitions, given that they do not justify retribution. I draw a parallel from recent work on implicit biases to make a case for taking moral responsibility for retributive intuitions. In the same way that we can exert some form of control over our unwanted implicit biases, we can and should do so for unjustified retributive intuitions in cases of robot harm.

## Introduction

Our lives are increasingly affected by robotization. Recent developments in robotics and machine learning are initiating a “new generation of systems that rival or exceed human capabilities” (Kaplan 2015, 3). Advances in technology of this kind, for example in the form of intelligent military robots (Hellström 2013; Sparrow 2007), industrial robots (Solaiman 2017), or self-driving cars (Nyholm and Smids 2016), have far-reaching moral, social, and legal consequences. They generate philosophical questions, such as whether robots can be held morally and legally responsible for their actions (Gunkel 2017; Matthias 2004). These questions become especially pressing when something goes wrong, such as in the recent case of Elaine Herzberg, who was killed by a self-driving car in Arizona in 2018. We are at an early stage of understanding human responses to the (potential) products and effects of extensive robotization; yet it is vital that work in this area keeps up with technological advances and properly captures their significance.

John Danaher (2016) has recently examined some of the implications of advanced and widespread robotization for our attitudes toward punishment and blame in the face of perceived wrongdoing in cases involving robots with high degrees of autonomy. He warns that a retribution gap may open up when the human desire for retribution is confronted with a lack of appropriate subjects for retributive blame.^1^ When robots cause harm, human beings are wont to seek out culpable targets in order to exact retribution—yet none may be found. The potential social and moral implications of a retribution gap are substantial; it may lead to moral scapegoating and could even threaten the rule of law if legal systems fail to accommodate common intuitions. In the wake of Herzberg’s death, attacks were reported on self-driving cars in apparent retaliation. In a city near Phoenix, people threw rocks at a number of self-driving vans that were being tested; they also slashed their tires and generally pestered them, for instance by driving right in front of them and suddenly braking hard (Romero 2018).

In this paper, I scrutinize several components of the retribution gap argument, focusing on those pertaining to retributive intuitions in relation to normative claims. The “awkward dance,” as Danaher describes it (2016, 302), between descriptive psychology and normative ethics is what is most interesting and at the same time most controversial in the retribution gap discussion. I critically examine the so-called dance, especially in light of debunking work by Wiegman (2017) that targets deontological intuitions. I show that deontological intuitions, which are “intuitions that are revealed by widespread tendencies to judge or act [independently of] an act-consequential evaluation of actions”^2^ (193), are ultimately at work in Danaher’s account.

Empirically informed moral philosophy has witnessed a number of attempts to undermine the evidentiary status of moral intuitions (e.g., Kelly 2018; Nichols 2014). If retributivist intuitions and the blame-seeking to which they give rise are in fact deontological intuitions, as I argue they are, then one should ask what debunking those intuitions would mean for the retribution gap. I contend that the most pressing and morally significant gap in fact arises between retributive intuitions and *what one ought to do with them*, rather than between those intuitions and the unsuccessful attempt to find appropriate targets for blame in the case of robot wrongdoings.

When retributive intuitions are properly understood in relation to normative theory, there may be no retribution gap to speak of. Instead, the fundamental question becomes *what to do* with retributive intuitions in cases of robot harm where there are no eligible targets for retributive blame. In response to this question, I draw a parallel from recent work on implicit biases and moral evaluation to make a case for taking moral responsibility—by exerting ecological control—for retributive intuitions in cases of robot harm without clear candidates for moral blame. For if these retributive intuitions are unjustified, as I show they are, then one must not act on them.

## The Retribution Gap

In order to understand what the debunking of deontological intuitions means for the retribution gap, one must first be clear about how, precisely, the gap emerges. Danaher’s (2016) argument for the retribution gap may be summarized as follows:

1. Human beings are innate retributivists; when we perceive harm, we seek to identify and desire to punish a culpable wrongdoer. This (i.e., retribution) is regarded by many moral philosophers as the right theory.
2. As robotization becomes more ubiquitous in society, and when robots come to have high degrees of autonomy, it is likely that these robots will cause more harm than they have heretofore done.
3. People will be seeking targets for retributive blame in these cases.
4. It is unlikely that either the robots or their makers will be eligible for retributive blame.^3^
5. Therefore, a retribution gap arises: people want retributive punishment, but they fail to find an appropriate subject for the satisfaction of this desire.
6. Thus, increased robotization will lead to a retribution gap.

It is beyond the scope of this paper to examine in detail each of the six claims. I will assume that, aside from the relation between description and prescription that I wish to critically examine (as found primarily in claims 1, 3, and 5), the other claims hold true—the dynamics are as described by Danaher (2016).

The first claim in the argument is that people are innate retributivist, for which there is a substantial body of evidence (e.g., Carlsmith and Darley 2008; Jensen 2010). This is uncontroversial. What is not clear, however, is the nature of the retributivism with which Danaher is concerned. He defines retributivism as “the belief that agents should be punished, in proportion to their level of wrongdoing, because they deserve to be punished,” and distinguishes this from retributive blame, which is “appropriate when the agent is morally culpable for the harm that occurred” (2016, 302). He writes of “powerful psychological drives pushing people to locate subjects of retributive blame,” and a “general social desire for retribution” (ibid.).

What Danaher appears to be concerned with, and what is the most natural starting point in a discussion of people’s desires for retribution, are intuitions—more specifically, retributive intuitions. Danaher refers to intuitions directly only once in his paper,^4^ when he acknowledges that doctrines “can sometimes fail to comply with intuitions of retributive justice” (2016, 307). The psychological nature and process of retributive blame is relatively underdeveloped in Danaher’s account. The most straightforward explanation of the phenomenon would be that, upon learning of some committed harm, a retribution intuition arises in an agent. This intuition may then drive the agent to respond with some act of retribution—whether by their own hand or indirectly, for instance through others. With retributive intuitions identified as the starting point and proper domain for the forces that contribute to the retribution gap, it only remains to be said that retributive intuitions generally are a species of deontological intuitions (Wiegman 2017).

Danaher is therefore concerned with deontological intuitions, to the extent that retributive intuitions contribute to the retribution gap. Although he does not explicitly classify retributive intuitions as deontological intuitions, his working definition of retributivism (and underlying intuitions) as “the belief that people should be punished because they deserve it” (Danaher 2016, 305) contrasts squarely with consequentialist theories of punishment (and underlying intuitions) that are typically more forward-looking by stressing beneficial outcomes like deterrence (Greene and Cohen 2004).^5^ Given that deontological intuitions are “intuitions that are revealed by widespread tendencies to judge or act [independently of] an act-consequential evaluation of actions” (Wiegman 2017, 193), these must be involved in Danaher’s account of the retribution gap. For, without eligible targets for retributive blame, how could retributive intuitions pertain to the consequences of punishment? It is implausible that these retributive intuitions would inherently involve evaluations of actions in terms of consequences, when a potential consequences-bearing target is missing.^6^

The self-confessedly problematic aspect of Danaher’s first claim is its second part. Here, he adds that “many moral and legal philosophers believe that this is the right thing to do” to the idea that when there is perceived causal responsibility for harm, people attach retributive blame (2016, 301). One would have expected the normative claim about retribution to appear at the very end of the overall argument, in order to give weight to the retribution gap as something that is not merely a description of people’s psychological responses to cases where harm has been caused that cannot facilely be attributed to an agent. Instead, the normative claim that retributivism is justified is affixed to the beginning of Danaher’s argument. That the two—retributive intuitions and the justification of retribution—can and should be separated is at the heart of my critique. I will return to this idea shortly.

One final point must be made, which is that focusing on the so-called descriptive-prescriptive dance does not cut arbitrarily into the argument for a retribution gap. Danaher writes that “the combination of this normative stance with the general social desire for punishment is what makes the retribution gap worthy of our attention,” and, rather more forcefully, that it is “the potential mismatch between the general desire for retribution and the specific requirements of retributive moral theory” that makes the retribution gap “so disturbing” (2016, 302). It is crucial, therefore, that the reasoning behind this aspect of the account is sound.

Although it is not strictly the last, Danaher’s fifth claim—that if there are no appropriate subjects of retributive punishment to be found by retribution-seeking agents, a retribution gap emerges—does most of the work in the final stage of the argument.^7^ In relation to this fifth claim, Danaher is attentive to one potential criticism, which goes as follows. A moral retributivist believes that people should be punished because and when they deserve it; therefore, there cannot be a gap—for if there is no suitable target for retributive blame, then no one ultimately goes unpunished who deserves to be punished (2016, 305). Danaher grants that this view is correct, at least from the perspective of a retributivist, but insists that this does not mean that there are no moral or normative concerns that arise from the gap. He goes on to argue for three normative concerns related to the retribution gap, which “should be of interest to everyone” (ibid.). These are: (1) an increased risk of moral scapegoating, (2) a potential threat to the rule of law, and (3) a strategic opening for those who oppose retributivism. I will return to some of these implications later.

Since, as I have argued, deontological intuitions are at work in the formation of the retribution gap, it is important to assess their nature and meaning, particularly in relation to retribution as a normative theory.

## Debunking Retribution

Debunking arguments generally serve to “clear the epistemic ground: they show who owes a plausible justification for their beliefs in the second round of inquiry” (Sauer 2018, 3). The application of debunking arguments to moral theory has a relatively short but lively history (e.g., Kahane 2011; Singer 2005; Street 2006). I will focus on debunking within the purview of the kind of intuitions that I have argued underlie the retribution gap, namely deontological intuitions. On that subject, Greene (2008) set the stage with his dual-process theory of moral judgment, according to which consequentialist intuitions are produced by more cognitive processes, while deontological intuitions—including retributive intuitions—are produced by more emotional or ‘alarm-bell’ processes.^8^

Whether Greene’s dual-process theory allows him to successfully debunk deontology has been questioned (Berker 2009; Sauer 2012). A more promising approach is provided by Wiegman (2017), who extends the project of debunking deontological intuitions while building on Greene’s groundwork.^9^ His argument is intended to sever the evidentiary connection between a particular type of deontological intuitions—retributive intuitions—and the principles that it appears to support. In my view, Wiegman’s account has greater sophistication and viability than Greene’s, and is more directly translatable to the current discussion of the retribution gap.^10^

Instead of explaining the difference between deontological and consequentialist intuitions in terms of the difference between the relative influences of emotion and cognition,^11^ Wiegman (2017) captures the difference by distinguishing prospective from non-prospective processes, where non-prospective processes bestow non-derivative value on actions—that is, value which does not derive from the action’s consequences. He proposes the following debunking argument, which is worth quoting at length:

Suppose that some non-prospective processes were selected for their fitness-enhancing consequences. If these processes also cause deontological intuitions because of their consequences, then the function of non-prospective processes (producing good outcomes) disconnects them from the states of affairs that intuitions report (that actions have value aside from their outcomes). (201)

The evolution of non-prospective processes, therefore, gives us an undercutting defeater for deontological intuitions. That is, it provides an evidential defeating argument that dissolves the evidentiary link between psychological processes and the states of affairs that they represent.

In relation to retributive intuitions specifically, Wiegman offers anger as an example of a non-prospective process, since it places non-derivative value on actions. He delineates a retributivist principle R that is responsible for retributive intuitions:

R: The value (or justification) of an act of punishment is not (or not only) derived from the consequences of the act (or the practice) of punishment.

Wiegman then provides an evolutionary account of anger in relation to punishment and cooperation, in order to show that “deterrence (or any other consequence of punishment) cannot be an indicator of any value that a punishment might have aside from its consequences,” so that “the putative evolutionary function of anger in the production of retributive intuitions serves as an undercutting defeater for those intuitions with respect to R” (2017, 205). The evidentiary connection between retributive intuitions and the retributive principle R is thus severed.

Given that an emotion like anger produces retributive intuitions as a result of the biological consequences of those intuitions, the intuitions themselves turn out to be poor indicators of non-derivative value (cf. Street 2006). Deontological intuitions more generally may still have non-derivative value, but they cannot be used as (normative) evidence for or a justification of such value.

## Debunking the Retribution Gap

Now it is time to combine what has been said about deontological debunking and the retribution gap, in order to gauge what the former means for the latter.

What must be noticed first and foremost is that Wiegman’s principle R lines up neatly with Danaher’s conception of retribution and the consequences of retributive intuitions for cases of robot-caused harm. It follows from Wiegman’s account that deontological retributive intuitions in cases of robot harm are not a good indicator of the non-derivative value of punishment. One cannot derive normative conclusions from these intuitions.

Danaher is aware that the retribution gap as he describes it leaves room for non-retributive approaches to punishment. In one sense, then, Wiegman’s debunking account could be used to argue for just such an approach: retributive intuitions cannot be used as evidence for the non-derivative value of punishment, so that we ought to look elsewhere instead—to consequentialist theories of punishment, for instance.^12^ This is hasty, however. The retribution gap opens up precisely *because* of the interplay between descriptive psychological processes and normative theory, so that one cannot arrive at the ‘end’ of the gap after it has been opened up, so to speak, in order to then speculate—as if independently from the process itself—about part of what caused the gap in the first place, namely normative theory. Yet this is what Danaher appears to do.

What seems to be missing from the retribution gap account as it stands is what may be called a metaethical stance; a position from which to connect the descriptive to the normative in a more coherent way. Consider Danaher’s reference to the fact that there are many legal and moral philosophers who hold that the “retributive attitude” is correct (2016, 299). This broad statement of the normative status of retribution actually does very little work in Danaher’s argument for the retribution gap, which is perhaps surprising given the emphasis on the relation (“dance”) between descriptive and normative theory. All the same, in light of Wiegman’s debunking of deontological intuitions, it will not do to simply introduce normative conclusions concerning retribution, for there may be substantive differences in how those conclusions are reached. More specifically, if some of the arguments of the theorists that Danaher adduces rely on the ostensible evidentiary connection between retributive intuitions and the non-derivative value of punishment, then these, as I have shown, are inadmissible. Normative conclusions regarding the retributive attitude therefore cannot be assumed beforehand; they require careful argumentation and clarification when it comes to the role played by retributive intuitions in light of their evidential status.

It is beyond my scope here to dissect the arguments of the philosophers that are introduced by Danaher in support of the normative appropriateness of retribution. This would also give them more attention than Danaher does himself. Nevertheless, given that the blanket use of retributive intuitions as evidence for retributive theories of punishment has been shown to no longer be feasible, the case for a retribution gap will be better off without theories that rely on this logic. In any case, in the end, it is not clear what role—if any—normative theory plays in the retribution gap, for intuitions about retribution may lead to blame attribution and desire for punishment *entirely independently* of any formal normative theory of punishment. Purely descriptively, people may experience retribution intuitions in the case of robot harm, people may find no one specific to blame, and people may hence be left with an unsatiated retribution desire. In other words, normative theory does not play a causal role in the retribution gap.

If it is wrong to use retributive intuitions as evidence for non-derivative theories of punishment, then this also applies to cases of robot harm. Conversely, this means that the evidentiary fissure between retributive intuitions and normative theory is not just informative in cases of robot harm, but will extend to any instance where retributive intuitions are sought to be mapped onto particular states of affairs. Interestingly, Danaher writes that, if no appropriate subjects of blame are to be found in cases of robot harm, then “moral retributivists could respond [to this] by saying that ordinary folk simply need to recalibrate their intuitive judgments” (2016, 308). It is not clear why he singles out moral retributivists. If retributive intuitions cannot be used to support retributivism about punishment, then (1) everyone needs to recalibrate their intuitions, to the extent that they stand in an untenable relation to matters of fact, and (2) this is—or should be—as much of a concern to non-retributivists about punishment.

It takes a description of the retribution gap to be able to plug it. Nevertheless, if the biggest source of worry—the major form of risk—in cases of robot harm centers on an unanswered or unanswerable set of retributive intuitions, as I think it does,^13^ then what matters above all else is that these intuitions are addressed. Part of doing that means understanding their nature and the purpose they serve.

## Moral Responsibility

At the very least, retributive intuitions cannot be used to justify retributive punishment. Yet, although retribution is not justified by the retributive intuitions that people experience in cases of robot harm, those intuitions still remain. The discussion of the retribution gap, after all, is sparked by their presence and prevalence. This leaves us with the practical problem of what to do with them—the fundamental problem, as I understand it, behind the retribution gap.^14^ To be clear: retribution in cases of robot harm without an identifiable target for retribution might still be justified. It simply cannot be justified on the basis of retributive intuitions. One might wonder, then, what retributive intuitions are good for. If they merely point toward, but cannot justify, acts of retribution, then prudence would recommend that the intuitions be ignored—at least until they are deemed justified. For the ways in which people respond to cases of robot harm without clear targets for blame need to be justified, especially when this involves retribution. However, as soon as a theory of punishment is found to justify particular responses in these cases, retributive intuitions become superfluous. This is because there would be no good reason to act on one’s intuitions rather than to act according to the normative demands stipulated by a justified theory of punishment (cf. Unger 1996).

I want to take it one step further. Before I do so, it must be noted that worries about the retribution gap may turn out to be overblown, once we understand the agency of robots in terms of human–robot collaborations rather than as (purely) independent of human agency (Nyholm 2018a, b). Let us nevertheless assume that, as postulated by the retribution gap argument, there are and will be cases of robot harm where targets for moral blame are not to be found even though people search for them.^15^ It seems highly unlikely to me that retribution is the appropriate response in situations where there is truly no target for retributive blame. Danaher’s definition of retribution as “the belief that agents should be punished, in proportion to their level of wrongdoing, because they deserve to be punished,” and of retributive blame as “appropriate when the agent is morally culpable for the harm that occurred” (2016, 302) literally point toward nothing in cases of robot harm when there are no identifiable moral agents. This is, of course, part of the problem outlined by the retribution gap. If we combine the notion of pointing-to-nothing, however, with the idea that retributive intuitions cannot by themselves justify retribution, then we have at least a *prima facie* case against giving any weight to acting on retributive intuitions in the scenarios stipulated to give rise to a retribution gap.

The case for disregarding retributive intuitions is further strengthened by the fact that acting on them in cases of robot harm is likely to lead to morally wrong behavior like moral scapegoating.^16^ When the Arizonans attacked self-driving cars, one cannot say with certainty that what spurred them on were retributive intuitions. However, the case is suggestive.^17^ Consider the facts. Elaine Herzberg was struck and killed by a self-driving car operated by Uber; the human test driver (who was not operating the vehicle, but who was there to take control should this be necessary) was a woman named Rafaela Vasquez; and the car itself was a Volvo (Stilgoe 2019). The self-driving cars that the Arizonans sabotaged were operated by Waymo, a former Google project; the emergency backup drivers were, one must presume, not Rafaela Vasquez; and the cars themselves were different (not even the same model) from the one that struck Herzberg (Romero 2018). All in all, then, none of the relevant actors were the same across the two cases. More specifically, none of the eligible targets for moral blame—the operating company, the human drivers, or the car producers—were the same. Putting aside questions about the particular form that retribution took in this case,^18^ one is hard-pressed to find a justification for retribution here. In light of Danaher’s definition of retributivism as the belief that agents should be punished because and to the extent that they deserve it,^19^ it appears to me that none of the targets in the city near Phoenix were eligible candidates for moral blame. After all, none had a causal connection to the original accident. The case seems to illustrate the kind of moral scapegoating that can result from retribution gap dynamics.

To the extent that we are all subject to retributive intuitions in these cases (if the scope of the retribution gap is as wide as it has been described to be), it seems that we would do best not to yield to their influence. If I am right—if we ought to discount retributive intuitions in cases of robot harm without targets for moral blame—then this might seem like an uphill battle, insofar as intuitions are automatic, knee-jerk responses beyond conscious control (e.g., Greene 2008; Haidt 2001). It may appear that we are stuck with them and, relatedly, that we are not responsible for them. While intuitions—including those pertaining to retribution—appear to be beyond direct control, however, they need not be characterized as beyond *any* form of control. Railton (2014) has argued, for instance, that intuitions are part of a flexible and sophisticated learning system, which opens up the possibility of honing them over time in order that they may (better) guide decision and action. Even without such a neuroscientific approach, however, there are indirect ways in which retributive intuitions can be brought within the realm of individual control.

A parallel may be drawn here to recent work on implicit biases and moral character. Implicit biases are “discriminatory biases based on implicit attitudes or implicit stereotypes,” which are considered to be especially problematic because they tend to result in behavior that “diverges from a person’s avowed or endorsed beliefs or principles” (Greenwald and Krieger 2006). When characterized as unintentional, unavoidable, automatic associations, these biases look to be paradigmatically beyond an individual’s control (Holroyd 2012). Nevertheless, there are reasons to think that people are still morally responsible in some ways for their implicit biases (Holroyd et al. 2017). Holroyd and Kelly (2016) have built on the work of Andy Clark (2007; Clark and Chalmers 1998) to argue that we have ecological control over implicit biases, which is sufficient for moral evaluation. An individual takes ecological control “when they reflectively decide to manipulate their mental states or environment, so as to shape their cognitive processes” (Holroyd and Kelly 2016, 119). More precisely, what Holroyd and Kelly have in mind is:

…the recursive use of control to enhance and heighten control itself. An agent can do this by fine-tuning the role of subsystems which in turn help produce dispositions and behaviors that can better fulfil her more distal goals, thus allowing her to better behave in ways that more precisely reflect her intentions, and more crisply conform to her considered ideals and values. Ultimately, a person can calibrate subsystems that guide behavior until eventually they operate, on their own, in precisely the way she wants them to operate, even when she is not consciously and explicitly attending to them. (2016, 119)

One requirement of taking ecological control in this way is that an individual is at least sometimes able to reflectively control their behavior (Holroyd and Kelly 2016).

Retributive intuitions in cases of robot harm appear to me to be less elusive than the implicit biases targeted by Holroyd and Kelly (2016), and individuals are certainly capable (at least in principle) of reflecting on their intuitions and controlling their behavior. That is to say, if implicit biases are legitimate subjects for moral evaluation by virtue of being susceptible to ecological control by agents, then so are retributive intuitions. And if retributive intuitions are unjustified in cases of robot harm where there are no candidates for moral blame, then one ought not to let them guide one’s behavior. One must not be led by them to acts of retribution. Danaher writes that the implications of the retribution gap will vary “depending on your preferred theory of punishment” (2016, 307). I propose instead that what ultimately matters is the control that you exert over the retributive intuitions for which you are morally responsible.

One way of wielding ecological control over implicit bias is through implementation intentions. Implementation intentions are “if–then plans” that complement goal intentions by identifying “(a) a good opportunity to act,” and “(b) a suitable goal-directed response to that opportunity” (Webb et al. 2012, 15). Holroyd and Kelly offer the following example of how implementation intentions can be used to overcome an implicit bias: “[A]n individual seeking to exert control over her implicit biases might deliberately repeat to herself, ‘If I see a Black face, I will think ‘safe’,’ practicing this line of thought enough that it becomes routine and automatic, thus defeating her implicit racial bias” (2016, 122).

In the case of retributive intuitions, this approach might go as follows. The goal intention should be to disregard retributive intuitions in cases of robot harms without targets for blame. To this end, an individual might specify:

- “If I learn of robot harm but cannot identify a target for blame…”^20^
- “…then I will think that retribution is not the appropriate response.”^21^

Through implementation intentions, a strong link—a new association—may be created between the specified opportunities and responses, “so that the planned response ensues swiftly and effortlessly (i.e., relatively automatically) when the opportunity is encountered” (Webb et al. 2012, 15). This is a practical and feasible way, then, for one to take control over retributive intuitions, and it is my contention that one ought to do so. If retributive intuitions do not justify retribution in cases of robot harm without targets for moral blame, then one must not act on them.^22^ One way to avoid acting on them is to take ecological control of them. Recognizing that this is warranted is an important first step.

## Conclusion

I have argued that the retributivist intuitions underlying the retribution gap are properly understood as deontological intuitions, and I have applied a debunking argument to show that retributive intuitions cannot be used to justify retribution in cases of robot harm without eligible targets for moral blame. The upshot of this has been that, when it comes to robot harm, the most pressing gap in fact arises not between retributive intuitions and unsuccessful attempts to find appropriate targets for moral blame (as in Danaher’s account), but rather between retributive intuitions and how we act on them. The crucial task thus becomes to determine what we should do in light of the retributive intuitions we experience in cases of robot harm without clear candidates for moral blame. I have drawn a parallel from recent work on implicit biases and moral evaluation to suggest one potential course of action. Just as we should shoulder responsibility for our implicit biases, we ought also to take moral responsibility for our retributive intuitions. By exercising ecological control over them and forming implementation intentions, for instance, we can and must ensure that we do not engage in retribution on the basis of these unjustified intuitions.

I hope that there will be no more cases like Elaine Herzberg’s. Should similar events transpire in the future—that is, should robots with high degrees of autonomy cause harm—then retribution might be a morally appropriate response, perhaps even when there are no explicit targets for moral blame. But our retributive intuitions about them will not point us in the right direction.

## Footnotes

1 The retribution gap can be seen as a subcategory of responsibility gaps, which have been discussed at greater length in the literature, and which occur "when computerized, highly adaptive, autonomously operating devices, inevitably lead to a partial loss of the operator’s control over the device," (Matthias 2004, 176). Retribution and responsibility gaps, although related, are nevertheless supposed to be conceptually distinct (Danaher 2016).

2 I have slightly modified Wiegman's (2017) definition, here and elsewhere.

3 Much of Danaher's (2016) argument ultimately depends on this claim, which is not uncontested (see, e.g., Nyholm 2018a, b). It is clear that, if there are always eligible targets for retributive blame in cases of robot harm, then there will be no retribution gap. As systems become increasingly autonomous, complex, and more substantially embedded in society, however, it stands to reason that targets for retributive blame will accordingly become more obscure. I think that this is enough to warrant retribution gap concerns (for a defense of retribution-gaps along different lines, see also De Jong 2019).

4 Two other instances where he explicitly refers to intuitions involve explanations of the work of others.

5 This is the standard account of deontological versus consequentialist theories of punishment, where the two are set off against each other. In principle, however, there is no reason why consequentialist approaches could not accommodate deontological elements (e.g., those pertaining to the good or the right). The consequentialist could, for instance, include in their theory of the good a claim to the effect that it is good if evil people suffer (see also Portmore 2011). Thanks to Sven Nyholm for this point.

6 Of course, consequences may be taken as something larger and nonspecific here, for instance as consequences for society at large, should some form of retribution (without target) occur. This, however, seems to stretch retributive intuitions beyond recognition. It also goes against Danaher's (2016) point that, when it comes to the desire for retribution, people tend to want to see individuals punished. Thanks to an anonymous reviewer for pushing me to further develop this argument.

7 The sixth and last claim simply relates the fifth back to robotization.

8 Greene assumes that, within the domain of punishment, "deontological" and "retributivist" are essentially interchangeable, even though they are conceptually distinct (2008, 21). To my knowledge, equating the two in this way has not been challenged in the literature—at least not within the domain of punishment. This adds further weight to my claim that Danaher is concerned with deontological intuitions as much as with retributive intuitions.

9 Wiegman calls his account a "friendly amendment" to Greene's dual-process theory of moral judgment (2017, 194).

10 It must be noted that, while I have selected Wiegman's (2017) debunking account because it appears to me to be the most successful and pertinent, there may well be other ways to undermine retributive intuitions.

11 As, for instance, done by Greene (2008).

12 But see also footnote 2.

13 And Danaher appears to agree, in speaking of the "desire for retribution [that] will go unfulfilled" (2015, 305).

14 The argument that some (kinds of) moral intuitions are, upon further analysis, not justified—and therefore are not to be given weight in moral theory—is not new. Recently, for example, Greene (2013) has argued that deontological moral intuitions fail to track morally relevant features of moral dilemmas, so that one's moral theory—in Greene's case, a utilitarian metamorality—ought not to be based on them. (But see Kraaijeveld and Sauer (2019) for a critique of this approach.).

15 See also footnote 3.

16 Danaher writes that, "[i]f there is a deep human desire to find appropriate targets for retributive blame, but none really exist, then there is a danger that people will try to fulfill that desire in inappropriate ways. Or, perhaps even more serious than this, that other social actors will take advantage of the desire in inappropriate ways" (2016, 307).

17 I would like to thank an anonymous reviewer for suggesting that I discuss this case.

18 Attacking cars on the street, thus also putting human drivers and pedestrians at risk, is highly unlikely to be justified, even if some other form of retribution might be.

19 This is a fairly standard definition of retributivism (cf. Hanna 2018).

20 This 'if' may be further broken down into an 'if' and a 'then': "If I learn of robot harm… then I will try to identify a target for blame." Subsequently, "If I cannot identify a target…" will complete the sequence.

21 This is just one example of an if–then plan; it may be formulated in different ways and for different situations, as long as it reinforces the relevant overarching goal intention.

22 Retribution may still be justified in other ways, as I have previously pointed out; just not on the basis of retributive intuitions in these cases.
